# Interaction of interferon alpha therapy with thyroid function tests in the management of hepatitis C: a case report

**DOI:** 10.1186/s13256-016-1028-y

**Published:** 2016-09-15

**Authors:** Gurmit Gill, Hammad Bajwa, Peter Strouhal, Harit N. Buch

**Affiliations:** 1 Department of Endocrinology and Diabetes, New Cross Hospital, Wolverhampton, WV10 0QP UK; 2 Department of Radiology, New Cross Hospital, Wolverhampton, WV10 0QP UK

**Keywords:** Hepatitis C virus, Interferon alpha, Hyperthyroidism, Hypothyroidism, Autoimmune

## Abstract

**Background:**

Interferon alpha is a widely used therapeutic agent in the treatment of hepatitis C virus infection. Clinical thyroid disease is seen in nearly 15 % of patients receiving interferon alpha for hepatitis C virus infection. The mechanism of thyroid dysfunction with interferon alpha is either autoimmune or inflammatory. We report a case of young woman who developed biphasic thyroid dysfunction posing a diagnostic challenge, while receiving interferon alpha treatment for hepatitis C virus infection.

**Case presentation:**

A 29-year-old, Caucasian woman with type 1 diabetes and hepatitis C virus infection was referred with hyperthyroidism, while she was at 17 weeks of a planned 24-week course of interferon alpha therapy. A laboratory investigation revealed a thyroid stimulation hormone level of 0.005 mU/L (0.350–4.94), free thyroxine of 45.6 pmol/L (9.0–19.0) and free tri-iodothyronine of 12.6 pmol/L (2.6–5.7). She had a mild neutropenia and alanine aminotransferase at double the reference value. Her thyroid peroxidase antibody level was 497 ku/L (<5.6) and thyroid inhibitory factor 7 IU/L (>1.8 iu/l is positive). Thyroid scintigraphy with technetium99 scan confirmed a normal-sized thyroid gland with diffuse but normal overall uptake. A diagnosis of interferon alpha-triggered autoimmune hyperthyroidism as opposed to an inflammatory thyroiditis was made. She was offered radioactive iodine therapy, as thionamides were considered inappropriate in view of her liver disease and mild neutropenia. Due to our patient’s personal circumstances, radioactive iodine therapy was delayed by 8 weeks and her thyrotoxic symptoms were controlled with beta-blockers alone. A repeat thyroid function test, 4 weeks post treatment with interferon alpha, indicated spontaneous conversion to hypothyroidism with a thyroid stimulation hormone level of 100 mU/L, free thyroxine of 5.2 pmol/L and free tri-iodothyronine of 1.7 pmol/L. She subsequently received levothyroxine for 4 months only and had remained euthyroid for the last 3 months without any treatment.

**Conclusions:**

Initial investigations favored the autoimmune nature of hyperthyroidism but follow-up of the case, interestingly, was more consistent with inflammatory thyroiditis. We propose that this can be explained either on the basis of autoimmune subacute thyroiditis or a change in the nature of thyroid stimulation hormone receptor antibody production from stimulating-type to blocking-type antibodies, with disappearance of the latter on discontinuation of interferon alpha.

## Background

Interferon alpha is a widely used therapeutic agent and is employed in the treatment of hepatitis C virus infection (HCV) [1]. However, up to 15 % of patients receiving interferon alpha for HCV infection can develop clinical thyroid disease [2]. The precise relationship between interferon alpha therapy and the development of thyroid pathology can be complex and the mechanism of thyroid dysfunction should be assessed in each patient prior to initiation of treatment. We present a case where therapeutic decisions were difficult to make in view of the unusual course of thyroid dysfunction.

## Case presentation

A 29-year-old, Caucasian woman with HCV (genotype 3) was referred from a gastroenterology clinic with hyperthyroidism, diagnosed on routine thyroid function testing. She had a 23-year history of type 1 diabetes, which was complicated by nephropathy and proliferative retinopathy and for which she was on a multiple-dose insulin regime. She reported a 6-kg weight loss, heat intolerance, and amenorrhoea in the preceding 3 months. On examination, she had mild positional tremor but no other clinical features of hyperthyroidism. She did not have a palpable goiter or thyroid eye disease. At the time of referral she had been receiving interferon alpha therapy (180 mcg Pegasys® once weekly by subcutaneous administration) for 17 weeks of a planned 24-week course of treatment, in addition to 800 mg Copegus daily by oral administration.

**Fig. 1:**
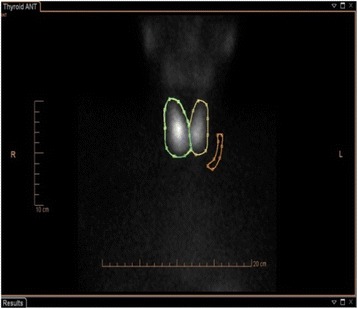
Radionuclide images

**Fig. 2:**
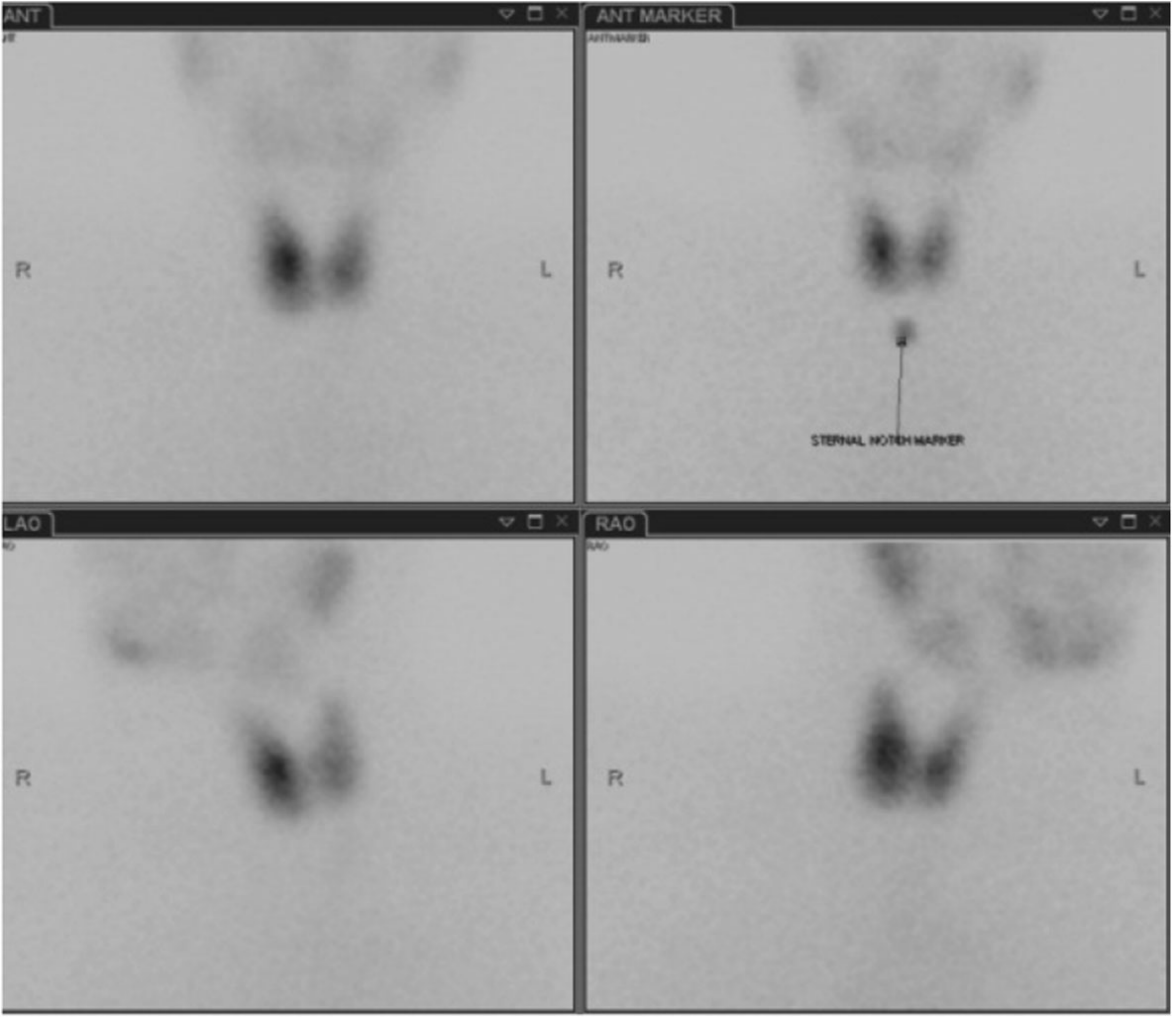
Radionuclide images

A laboratory investigation revealed a thyroid stimulation hormone (TSH) level of 0.005 mU/L (reference range 0.350–4.94), free thyroxine (FT_4_) of 45.6 pmol/L (9.0–19.0) and free tri-iodothyronine (FT_3_) of 12.6 pmol/L (2.6–5.7). Her hemoglobin was 127 g/L (115–165), white cell count 1.8 × 10E9/L (4.0–11.0), platelets 118 × 10E9/L (150–450) neutrophils 1.0 × 10E9/L (2.0–7.5), bilirubin 7 umol/L (0–20), alkaline phosphatase 63 iu/L (30–130), alanine transaminase 120 iu/L (<55), albumin 32 g/L (35–50) and she had normal renal function. Her thyroid peroxidase (TPO) antibody level was 497 ku/L (<5.6) and thyroid inhibitory factor was 7 IU/L (<1.0 iu/L is negative, 1.0–1.8 iu/L is borderline, >1.8 iu/L is positive). A thyroid scintigraphy with technetium99 scan (Radionucleotide (RN) scan) revealed a normal-sized thyroid gland with diffuse but normal overall uptake but not suppressed (Figs. 1 and 2).

On the basis of the above investigations she was suspected to have interferon alpha-triggered hyperthyroidism related to a diffuse autoimmune process as opposed to an inflammatory thyroiditis. In view of persistent hyperthyroidism over subsequent weeks and the likelihood of the autoimmune process to continue after withdrawal of interferon alpha, it was decided to initiate specific therapy to control hyperthyroidism. Thionamides were not considered appropriate in view of her history of liver disease and suppressed neutrophil count and a decision was made to administer radioiodine (RAI) therapy for which she was appropriately counselled. However, due to our patient’s personal circumstances, RAI therapy was delayed by 8 weeks and her hyperthyroid symptoms were controlled with beta-blockers. During this period she completed her 24-week interferon alpha therapy and when she presented for her pre-RAI assessment she had been off interferon for 4 weeks. A repeat thyroid function test indicated spontaneous conversion to hypothyroidism with a TSH level of 100 mU/L, FT_4_ of 5.2 pmol/L, and FT_3_ of 1.7 pmol/L. Repeat biochemical testing 3 weeks later confirmed persistent hypothyroidism and, in view of her significant symptoms, she was commenced on thyroxine replacement. She remained euthyroid on 100 mcg thyroxine over the next 4 months. At the end of this period thyroxine was withdrawn, and when last seen 3 months later she had remained euthyroid off thyroxine and was hepatitis C virus free.

## Discussion

Interferon alpha is the cornerstone of therapy for hepatitis C infection (HCV) [1, 3, 4]. It can achieve a sustained virological response in up to 50 % of patients and hence has been widely used as therapeutic agent in patients with HCV infection. However, new-onset thyroid dysfunction is a major side effect and prevalence rates of up to 15 % have been reported [2].

The mechanism of thyroid dysfunction with interferon alpha is either autoimmune or inflammatory [5]. In one series of 321 patients, of the 10 patients who developed hyperthyroidism, 6 had an autoimmune presentation while the other 4 patients had destructive thyroiditis [6]. Most other series report a fairly even distribution between the prevalence of the two types of thyroid dysfunction [7].

The diverse effects of interferon alpha on the immune system that leads to its therapeutic actions are also responsible for the development of autoimmunity. It binds to interferon alpha receptors, which are transmembrane glycoproteins-containing cytoplasmic domains. As a result it activates various signaling pathways, including the Janus kinase/signal transducer and activator of transcription (JAK-STAT) pathway, the proto-oncogene Crk (Crk) pathway, the insulin receptor signaling (IRS) signaling pathway and the mitogen-activated protein (MAP) kinase pathway, which in turn leads to activation of a large number of interferon-stimulated genes (ISGs) including cytokine and adhesion molecule genes [8–10]. Interferon alpha induced increased expression of MHC class 1 antigens on the thyroid cells is another possible mechanism of stimulating autoimmunity. Interferon alpha also shifts the immune response to a type 1 helper (Th1)-mediated pattern [11, 12], resulting in the production of potent pro-inflammatory cytokines including interferon gamma (IFN-γ) and interleukin-2 (IL-2), thereby stimulating autoimmunity [13, 14]. Other possible mechanisms include stimulation of interleukin-6 (IL-6) release, enhancement of the activity of neutrophils, lymphocytes, macrophages and natural killer (NK) cells, all of which can potentially contribute to development of autoimmunity-mediated inflammatory response [15, 16]. It has also been demonstrated from molecular studies that HCV proteins could independently impact and contribute to autoimmune thyroid dysfunction [17] through the binding of HCV glycoproteins to CD8 receptors expressed on thyroid cells and inducing a cascade of signaling pathways leading to interleukin-8 (IL-8) release.

Forty percent of patients who develop thyroid dysfunction while on interferon alpha therapy become thyroid antibody positive, and antibodies prior to initiation of interferon alpha therapy have a positive predictive value of 67 % for the development of thyroid dysfunction [7]. Patients who progress to hyperthyroidism demonstrate a significant rise in thyroid antibody levels [18]. This has led to the recommendation that thyroid antibody testing should be undertaken prior to initiation of interferon alpha therapy. Other predictors include female gender and lower FT_3_ levels [7, 19].

Inflammatory non-autoimmune thyroiditis is usually a destructive process occurring within the gland. It results from the direct effect of interferon alpha on thyroid cells with inhibition of TSH-induced gene expression of thyroglobulin, TPO, and sodium iodide symporter as shown in various laboratory studies [20].

The clinical course of autoimmune thyroid dysfunction is similar to that of Graves’ disease and patients require standard anti-thyroid management especially as, in most cases, withdrawal of interferon alpha does not lead to resolution of hyperthyroidism [6, 13, 21] and patients remain thyroid antibody-positive [6, 13, 21]. Use of anti-thyroid drugs has to be carefully considered in view of the hepatic dysfunction and neutropenia that accompanies the use of interferon alpha. On the other hand, inflammatory non-autoimmune thyroid dysfunction is clinically characterized by three phases, that is sudden onset of hyperthyroidism, followed by a hypothyroid state and eventually resolution and normalization of thyroid function, which can take several weeks to months [22]. As spontaneous resolution is expected, anti-thyroid therapy is not required for management of hyperthyroidism. Beta-blockers are used for symptomatic benefit and, during the hypothyroid phase, patients may require thyroxine with planned withdrawal after 3–6 months. Five percent of patients have permanent hypothyroidism and patients who have complete recovery remain susceptible to recurrence on being re-exposed to interferon alpha.

In view of its impact on the management strategy, it is important to identify the mechanism of interferon alpha-induced thyroid dysfunction with appropriate investigations. Thyroid antibodies, more specifically TSH receptor antibodies and radionuclide uptake scans are helpful in making this distinction. Positive antibodies with non-suppressed radionuclide uptake suggest an autoimmune process while negative antibodies with suppressed radionuclide uptake would support inflammatory thyroiditis.

In summary, the case emphasizes the need to assess the risk of development of thyroid dysfunction prior to commencement of interferon alpha in patients with HCV infection and to monitor such patients closely clinically and biochemically. It also highlights the importance of appropriate investigations to elucidate the possible mechanism of thyroid dysfunction prior to formulating management plans, although an unexpected pattern of thyroid dysfunction is occasionally encountered, as in our patient.

## Conclusions

Our patient had an unexpected course of hyperthyroidism. She had several features to suggest an autoimmune process with her background of autoimmune type 1 diabetes; positive TSH receptor antibodies and a non-suppressed radionuclide uptake scan, undertaken during significant hyperthyroidism. While waiting for definitive management in the form of RAI therapy, she spontaneously reverted to hypothyroidism, which was transient, and our patient eventually staged a full recovery of thyroid function. This sequence of thyroid dysfunction was suggestive of destructive thyroiditis rather than diffuse autoimmune hyperthyroidism. The explanation for the discrepancy between investigation results and the clinical course was unclear. Positive antibodies and biphasic thyroid dysfunction can be explained on the basis of autoimmune subacute thyroiditis. However this is not supported by radionuclide scan, which would be expected to show suppressed uptake in inflammatory thyroiditis of any etiology. A change in iodine status has been linked to fluctuating and varying thyroid status in patients on interferon alpha [23]. However our patient had been residing in the UK for a number of years and there were no other factors to suspect this possibility. A more likely explanation is a change in the nature of antibody production from stimulating-type to blocking-type antibodies, with disappearance of the latter on discontinuation of interferon alpha. This was difficult to confirm, as assays for identifying subtypes of TSH receptor antibodies are not readily available. The changing nature of antibodies has been observed anecdotally in other situations of induced autoimmune process. A similar course was also reported in a series of four patients following the use of alemtuzumab for multiple sclerosis, with one of the patients developing hypothyroidism soon after the initial presentation with hyperthyroidism [24].

## Abbreviations

Crk: Proto-oncogene Crk
FT_3_: Tri-iodothyronine
FT_4_: Free thyroxine
HCV: Hepatitis C virus
IFN-ɣ: Interferon gamma
IL-2: Interleukin-2
IL-6: Interleukin-6
IL-8: Interleukin-8
IRS: Insulin receptor signaling
ISGs: Interferon-stimulating genes
JAK-STAT: Janus kinase/signal transducer and activator of transcription
MAP: Mitogen-activated protein
NK: Natural killer
RAI: Radioiodine
Th1: Type 1 helper cells
TPO: Thyroid peroxidase
TSH: Thyroid-stimulating hormone
